# Identification of long noncoding RNAs involved in plumule-vernalization of Chinese cabbage

**DOI:** 10.3389/fpls.2023.1147494

**Published:** 2023-03-14

**Authors:** Yun Dai, Guoliang Li, Xinyu Gao, Shaoxing Wang, Ze Li, Chao Song, Shifan Zhang, Fei Li, Zhiyuan Fang, Rifei Sun, Hui Zhang, Shujiang Zhang

**Affiliations:** ^1^ Institute of Vegetables and Flowers, Chinese Academy of Agricultural Sciences, Beijing, China; ^2^ State Key Laboratory of Vegetable Biobreeding, Institute of Vegetables and Flowers, Chinese Academy of Agricultural Sciences, Beijing, China

**Keywords:** plumule-vernalization, lncRNAs, mRNAs, ceRNAs, shoot apical meristem, FLCs

## Abstract

Vernalization is a phenomenon in which plants must undergo a period of continuous low temperatures to change from the vegetative growth stage to the reproductive growth stage. Chinese cabbage is a heading vegetable, and flowering time is an essential developmental trait. Premature vernalization leads to premature bolting, which causes a loss of product value and yield. While research into vernalization has provided a wealth of information, a complete understanding of the molecular mechanism for controlling vernalization requirements has not yet been elucidated. In this study, using high-throughput RNA sequencing, we analyzed the plumule-vernalization response of mRNA and long noncoding RNA in the bolting-resistant Chinese cabbage double haploid (DH) line ‘Ju Hongxin’ (JHX). A total of 3382 lncRNAs were identified, of which 1553 differentially expressed (DE) lncRNAs were characterized as plumule-vernalization responses. The ceRNA network revealed that 280 ceRNA pairs participated in the plumule-vernalization reaction of Chinese cabbage. Through identifying DE lncRNAs in Chinese cabbage and analyzing anti-, cis-, and trans-functional analysis, some candidate lncRNAs related to vernalization promoting flowering of Chinese cabbage and their regulated mRNA genes were found. Moreover, the expression of several critical lncRNAs and their targets was verified using qRT-PCR. Furthermore, we identified the candidate plumule-vernalization-related long noncoding RNAs that regulate *BrFLCs* in Chinese cabbage, which was interesting and different from previous studies and was a new discovery. Our findings expand the knowledge of lncRNAs in the vernalization of Chinese cabbage, and the identified lncRNAs provide rich resources for future comparative and functional studies.

## Introduction

Flowering time is significant for harvesting valuable agricultural products. Exposure to low temperatures causes plants to bolt and flower, especially in *Brassica* green leafy vegetables, which are sensitive to vernalization. Bolting and flowering lead to the devaluation of agricultural products, and breeding high bolting resistant varieties has important economic significance. In addition, the control of flowering time is crucial for crop seed breeding, which can rapidly breed seeds and obtain high-purity materials. Chinese cabbage (*Brassica rapa* L. ssp. *pekinensis*) is a cruciferous leafy *Brassica* vegetable that originated in China and has a long history of cultivation (Dai et al., 2021). The vernalization of Chinese cabbage can be divided into two types: seedling-vernalization and plumule-vernalization (Yu et al., 2016). Seedling-vernalization is a kind of vernalization in which seedlings grow to a certain size, accumulate certain nutrients, and then experience low-temperature treatment; plumule-vernalization is a kind of vernalization mode in which seeds are subjected to low-temperature treatment in the germination state (Yu et al., 2016).

The second step of the central dogma is to translate genetic information from RNA to protein, which is crucial. However, more than 75% of RNA transcripts are not translated into proteins, which does not mean they have no function (Zhang et al., 2019). Among them, long noncoding RNA (lncRNA) is a noncoding RNA with a length of more than 200 nt (Bhat et al., 2016). Although lncRNA is a transcript with relatively low abundance, it has many functions and regulatory roles in transcriptional, post-transcriptional, and epigenetic aspects (Dykes and Emanueli, 2017; Zhang and Chen, 2017). LncRNAs form complementary double strands with transcripts encoding protein genes, interfering with mRNA shearing to form different shear forms, and they bind to specific proteins. LncRNA transcripts can regulate the activity of the corresponding proteins and act as a structural component to form nucleic acid–protein complexes with proteins, etc. (Lam et al., 2014). With the development of sequencing technology, an increasing number of lncRNAs have been identified in many crops, such as *Arabidopsis thaliana* (Lu et al., 2017), *Solanum lycopersicum* (Wang et al., 2018a), *Solanum tuberosum* (Kwenda et al., 2016), *Oryza sativa* (Yuan et al., 2018; Wang et al., 2018b), and *Zea mays* (Li et al., 2014). Currently, some related lncRNAs have also been studied in various characteristics of Chinese cabbage. In a downy mildew study of Chinese cabbage, disease-responding mRNAs and long noncoding RNAs were identified using two resistant lines and one sustainable line (Zhang et al., 2021). In addition, there have been studies on lncRNAs in Chinese cabbage under heat stress. Song et al. conducted a comparative analysis of 37 lncRNAs, comprehensively analyzed lncRNAs in Chinese cabbage, and expanded some lncRNAs identified in the Chinese cabbage heat stress response (Song et al., 2021). Research on lncRNAs in Chinese cabbage is still scarce and requires further analysis.

In recent years, lncRNAs about plant flowering have been increasingly reported. LncRNA *MAS* fine-tune flowering time by regulating the expression of *MAF4*, a member of FLOWERING LOCUS C (*FLC*) family (Zhao et al., 2018); Intronic long noncoding RNA, RICE FLOWERING ASSOCIATED (*RIFLA*) regulates *OsMADS56*-mediated flowering in rice (Shin et al., 2022); Genome-wide was used to screen and identify some long non-coding RNA related to flowering/bolting of *Lactuca sativa* (Soorni et al., 2023). FLC is a flower inhibitor that can maintain the vegetative growth of plants. Once plants experiencing a long period of cold, *FLC* activates several important genes in the process of plant growth by inhibiting a series of genes required to activate the transition from apical meristem to inflorescence and finally converts the vegetative growth of plants into reproductive growth (Michaels and Amasino, 1999; Whittaker and Dean, 2017), and its related lncRNAs have also been a research hotspot. There are three main types of lnRNAs involved in the vernalization of Arabidopsis: cold-induced long antisense intragenic RNA (*COOLAIR*), which encompasses most of the FLC locus from 5’ to 3’ polyadenylation sites, and *COOLAIR* is alternately involved in polyadenylation and splicing (Liu et al., 2010; Sun et al., 2013b); COLD ASSISTED INTRONIC NONCODING RNA (*COLDAIR*) has been identified in the first intron of *FLC* in the sense direction (Kim et al., 2017b); and *COLDWRAP* (cold of winter-induced noncoding RNA from the promoter) overlaps with the promoter region of *FLC* (Kim and Sung, 2017a). These three lncRNAs are affected by vernalization and silence the expression of *FLC*, thus promoting the reproductive transformation of *Arabidopsis*. In the study of vernalization-related lncRNAs in Chinese cabbage, only *COOLAIR*-like transcripts have been detected in *BrFLC2* (Li et al., 2016), and thus far there are no reports on *COLDAIR* or *COLDWRAP* transcripts in Chinese cabbage.

In this study, we first conducted plumule-vernalization of different degrees on a bolting-resistant Chinese cabbage DH line, determined the sequencing period of RNA-seq according to the phenotypes of the shoot apical meristem (SAM) section and the flowering time after vernalization, and characterized lncRNAs related to Chinese cabbage vernalization. A total of 3382 lncRNAs were identified in response to Chinese cabbage plumule-vernalization. Anti-, cis-, and trans-functional analysis of DE lncRNA revealed that some lncRNAs related to vernalization promoted flowering in Chinese cabbage. The structure and expression of antisense lncRNAs regulating *BrFLC* were further studied to reveal the molecular mechanism of Chinese cabbage plumule-vernalization.

## Materials and methods

### Plant material and vernalization treatment

**Figure 1:**
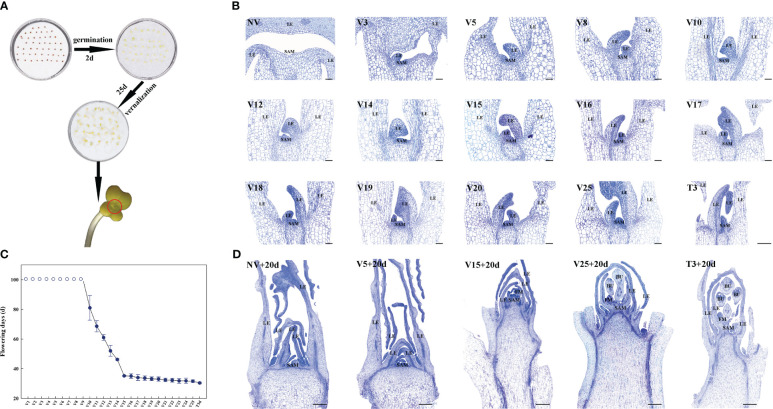
Plumule-vernalization and toluidine blue staining of Chinese cabbage to identify RNA-seq samples (short scale bar, 500 μm; long scale bar, 1000 μm). **(A)** Brief process of germination and plumule-vernalization of Chinese cabbage. **(B)** Toluidine blue staining of meristems in different plumule-vernalization periods (SAM: shoot apical meristem; LE: leaf or leaf primordia). **(C)** The number of flowering times in different plumule-vernalization periods after sowing (the hollow circle represents more than 100 days of flowering). **(D)** Toluidine blue staining of meristems in different RNA-seq samples after 20 days of sowing (SAM: shoot apical meristem; LE: leaf or leaf primordia; FM, floral meristem; BU: bud).

The bolting-resistant Chinese cabbage DH line ‘Ju Hongxin’ (JHX) was provided by the Institute of Vegetables and Flowers of the Chinese Academy of Agricultural Sciences located in Beijing, China. We have slightly modified the Chinese cabbage seed treatment method of Yu et al., and have conducted germination and plumle-verilization treatment for ‘JHX’ (Yu et al., 2016). Full and uniform seeds of ‘JHX’ were selected, rinsed with sterilized water, and then placed in petri dishes covered with a double layer of filter paper. The seeds were placed in a climate chamber at a constant temperature of 25°C and 16 h/d light for two days to accelerate germination. After the radicle broke through the seed coat, the petri dishes were moved to a vernalization chamber at 4°C (22/2 h light/dark photoperiod and 150 µmol m^−2^ s^−1^ light intensity) for 25 days (**Figure 1A**) and then recovered at 25°C (22/2 h light/dark photoperiod and 150 µmol m^−2^ s^−1^ light intensity) for 3 days. Samples under different treatments were collected, and all samples were immediately frozen in liquid nitrogen and stored at −80°C temperature freezer.

### Toluidine blue staining and flowering time analysis

The SAM of ‘JHX’ grown under 4°C and 25°C conditions were harvested at different time points after the vernalization and recovery treatments and placed in formaldehyde/acetic acid/ethanol fixative solution. After harvesting, the AFF fixing solution (Beijing Solarbio Science & Technology Co., Ltd., Beijing, China) and dehydration system (JJ-12J; WHJJ, Wuhan, China), embedding system (JB-P5; WHJJ, Wuhan, China), and rotary slicer (LeicaRM2016; Leica, Shanghai, China) were used to fix, dehydrate, embed, and slice the shoot apexes. Sections of 8 μm thickness were placed on glass slides, baked in an oven at 60°C, and stored at room temperature for standby after baking. The sections were dewaxed and stained with toluidine blue dye solution, as described previously (Wahl et al., 2013). Samples after different plumule-vernalization times were conventionally planted at 25 ± 2°C (16/8 h light/dark photoperiod and 150 µmol m^−2^ s^−1^ light intensity). The flowering times were recorded when the first flower opened.

### RNA extraction

The 1g of each sample taken from the −80°C temperature freezer and ground into powder, and total RNA was extracted from each sample using Trizol kit (Invitrogen, Carlsbad, CA, USA), and then DNase I (Takara Bio, Dalian, China) was used to remove contaminated genomic DNA (gDNA). The extracted RNA was subjected to agarose gel electrophoresis to check the integrity of RNA samples, and the OD value of RNA was detected by NanoDrop (Thermo Scientific, United States) to determine the purity of RNA. After passing the quality detection, RNAs were utilized to construct libraries.

### Library construction and sequencing

The overall process of library construction includes remove ribosome RNA (rRNA), RNA fragmentation (200-500nt), first strand cDNA synthesis, ligata adapter, UNG (Uracil-N-Glycosylase) treatment and PCR amplification. Total 1.5 µg RNA from each sample was treated with the Epicentre Ribo-zero™ rRNA Removal Kit (Epicentre, USA) to retain mRNAs and ncRNAs. Subsequently, RNA sequencing libraries were constructed using the NEBNextR UltraTM Directional RNA Library Prep Kit for IlluminaR (NEB, United States) according to the manufacturer’s recommendations. Firstly, total RNA was fragmented with divalent cations in NEBNext First Strand Synthesis Reaction Buffer (5X) by increasing temperature. M-MuLV Reverse Transcriptase (RNase H-) and random hexamer primers were used to prepare first-strand cDNA, and second-strand cDNA were synthesized by DNA polymerase I, RNase H, dNTP (dUTP instead of dTTP) and buffer. Next, the cDNA fragments were purified with QiaQuick PCR extraction kit (Qiagen, Venlo, The Netherlands), end repaired, poly(A) added, and ligated to Illumina sequencing adapters. Then UNG was used to digest the second-strand cDNA. The second-strand of cDNA was digested at 37 °C for 15 minutes, and then at 95 °C for 5 minutes. The digested products were size selected by agarose gel electrophoresis, PCR amplified. Index (X) Primer, Universal PCR primers, and Phusion High-Fidelity DNA polymerase were used for the PCR, and the PCR product was purified using AMPure XP Beads (1.0X). Before constructed the library, used the library quality inspection kit DNA 1000 assay Kit (Agilent Technologies, 5067-1504) to test the quality of the library to ensure that the quality of the library was qualified. Finally, the Illumina HiSeq™ 4000 by Gene Denovo Biotechnology Co. (Guangzhou, China) was used for sequencing.

### LncRNA and mRNA identification

Raw reads obtained from RNA sequencing were first processed to remove adapters and low-quality reads. An index of the reference genome was built, and clean paired-end reads were mapped to the Chinese cabbage genome version 3.0 (http://brassicadb.cn) using HISAT2 version 2.1.0 (Kim et al., 2015) and other parameters set as defaults. The reconstruction of transcripts was carried out with Stringtie software version 1.3.4 (Pertea et al., 2015; Pertea et al., 2016). The final transcripts were generated with the Cuffmerge tool (Trapnell et al., 2010), and the transcripts obtained from each sample were merged. LncRNAs were identified according to the method described by Wang et al. (Wang et al., 2017). The length of the transcript was longer than 200 bp, and the exon number was greater than 1. The coding potential of the transcripts was evaluated using coding-noncoding index (CNCI) (Sun et al., 2013a) and Coding Potential Calculator (CPC) (Kong et al., 2007) programs.

### Analysis of differentially expressed mRNAs and LncRNAs

For each transcription region, the FPKM (fragment per kilobase of transcript per million mapped reads) values were calculated using StringTie software to quantify expression abundance and variation (Pertea et al., 2015). Differentially expressed transcripts of coding RNA and lncRNA were analyzed. DESeq2 software (Love et al., 2014) was used to analyze the differential expression of RNA and lncRNA between two different groups, and edgeR (Robinson et al., 2010) was used to analyze the differential expression of RNA and lncRNA between the two samples. Genes with a false discovery rate (FDR) < 0.05 and absolute fold change ≥ 2 were considered differentially expressed genes (DEGs). To evaluate the lncRNA expression patterns of Chinese cabbage in vernalization, STEM software (Ernst and Bar-Joseph, 2006) was used to cluster the 1553 DE lncRNAs based on their expression patterns.

### LncRNA–mRNA association analysis and gene functional annotation

Antisense lncRNAs may regulate gene silencing, transcription, and mRNA stability. RNAplex software (Tafer and Hofacker, 2008) was used to predict the complementary correlation between antisense lncRNA and mRNA. Upstream lncRNAs with a promoter or other cis-elements may regulate gene expression at the transcriptional or post-transcriptional level. The downstream or 3’UTR regions of lncRNAs may have other regulatory functions. Therefore, lncRNAs less than 100 kb upstream/downstream of the gene may be cis-regulators. Another function of lncRNAs is to trans-regulate co-expressed genes that are not adjacent to the lncRNA. The lncRNA–mRNA pairs were considered co-expressed when the Pearson correlation coefficient was more than 0.999, and the mRNA was predicted to act in trans on the corresponding lncRNA genes. Then, the GO (Young et al., 2010) and KEGG pathways (Mao et al., 2005) of the target genes were enriched and analyzed. To identify critical lncRNAs associated with vernalization, an interaction network comprising DE lncRNAs and DE mRNAs was constructed using Gephi (v0.8.2) software (Jacomy et al., 2014) based on anti-, cis-, or trans-regulation.

### Interaction network and ceRNA network construction

Chinese cabbage mature miRNA sequences were downloaded from miRBase (Kozomara et al., 2019). DE lncRNA and mRNA co-expression were used to construct ceRNA prediction libraries and target mRNAs of miRNAs, respectively. The ceRNA of Chinese cabbage miRNA was predicted using the RNAhybrid program (Krüger and Rehmsmeier, 2006), and psRNATarget program (Dai and Zhao, 2011) was used to predict he target mRNA of miRNA. The LncRNA–miRNA–mRNA network was subsequently constructed using Cytoscape v3.7.2 software (Shannon et al., 2003).

### Real-time quantitative PCR validation of lncRNAs and mRNAs

The candidate lncRNAs and their potential target genes were selected for qRT-PCR analysis to verify the sequencing results of the lncRNAs. First, the extracted RNA was reverse-transcribed into cDNA using HiScript III All-in-One RT SuperMix Perfect (Vazyme, Nanjing, China), and Taq Pro Universal SYBR qPCR Master Mix (Vazyme, Nanjing, China) was added. The CFX-96 Real-time System (BIORAD, Hercules, CA, USA) was used for qRT-PCR. Primer v5.0 was used for primer design (**Supplementary Table 1**). *Actin* was considered the internal control, and the gene expression data were analyzed using the 2^−ΔΔCt^ method (Livak and Schmittgen, 2001). SPSS v19.0 (SPSS, Chicago, IL, USA) was used to conduct one-way analysis of variance (ANOVA) with Duncan’s multiple range *post hoc* test, and there was a significance threshold of *p* < 0.05. The results were visualized using Sigmaplot v10.0 (Systat Software Inc., San Jose, CA, USA).

## Results

### Phenotype identification to select appropriate samples for RNA-seq

The ability of plants to produce new organs is due to the lifelong maintenance of the population of pluripotent stem cells in a specialized reservoir called the SAM. To more accurately identify and explore the plumule-vernalization response of lncRNAs and the regulatory mechanism in Chinese cabbage, we sliced the SAM under different plumule-vernalization treatments and different flowering times after plumule-vernalization (**Figure 1C**), stained the samples with toluidine blue (**Figure 1B**), and selected appropriate samples for RNA-seq. The SAM also grew with the progress of plumule-vernalization, while the leaves (LE) grew from non-vernalization (NV) to 25 days of vernalization (V25) (**Figure 1B**). The sown seeds sprouted after different plumule-vernalization times, and their flowering time (10 repeats at each period) was calculated. The flowering time was more than 100 days from V1 to V9 and 81–35.2 days from V10 to V15 (**Figure 1C**). After V15, flowering lasted until V25, and the flowering time was stable from 31.6 to 35.2 d. The longer the plumule-vernalization time, the shorter the flowering time. Seed at V25 was recovered after 3 days at 25°C and then planted; its flowering time was 30.4 d, indicating that the temperature recovery did not reduce the effect of vernalization. NV was the control sample; V5 referred to the sample that had experienced vernalization but had not achieved the effect of vernalization; V15 was the sample with the earliest flowering after the shortest vernalization time; V25 was the saturated vernalization sample; and T3 was the recovery sample after vernalization. We planted the seeds during these five periods. After 20 days, the shoot apical of the seedlings was collected for sectioning and dyeing (**Figure 1D**). SAM at NV+20d and V5+20d did not protrude, but in V15+20d, the shoot tips developed rapidly and produced the floral meristem (FM). At V25+20d and T3+20d, the shoot tips not only developed rapidly but also produced the FM and bud (BU). These phenotypes were consistent with the results of the SAM slice and flowering time after plumule-vernalization. Finally, we decided to use these five periods, with three biological replicates each, for a total of 15 RNA-seq samples.

### Identification and characterization of lncRNAs in Chinese cabbage during plumule-vernalization

Fifteen samples (three replicates for each sample) were collected for transcriptome sequencing and analysis. We obtained more than 60 million clean reads from each sample, of which 83.76–85.56% were mapped to the Chinese cabbage genome (version 3.0), and 79.42–81.90% of the clean reads in each sample were mapped to a unique gene (**Supplementary Table 2**). Using Pearson correlation analysis on these samples based on the FPKM values of mRNA and LncRNA of all transcripts, a strong correlation was found between the three biological repeats (**Supplementary Figures 1A, C**; **Supplementary Table 3**). Principal component analysis (PCA) showed that V15 and V25 samples had a high compactness (**Supplementary Figures 1B, D**), which was consistent with the SAM observation and flowering time, indicating that the plumule-vernalization effect of V15 to V25 may be similar. In total, 45,595 mRNA transcripts were obtained from 15 libraries, including 842 novel transcripts; 3382 lncRNA transcripts were obtained (**Supplementary Table 4**). The DEGs were obtained from comparisons between samples at V5, V15, V25, and T3 and those at NV (listed in **Supplementary Table 5**). NV vs. T3 had the most DEGs (8708 DEGs), followed by NV vs. V25 (7805 DEGs) and NV vs. V15 (6829 DEGs). Although there was no significant difference between V15 and V25 in the SAM phenotype and flowering time, there were still about 1000 DEGs between them. The longer the plumule-vernalization time, the more other genes changed.

**Figure 2:**
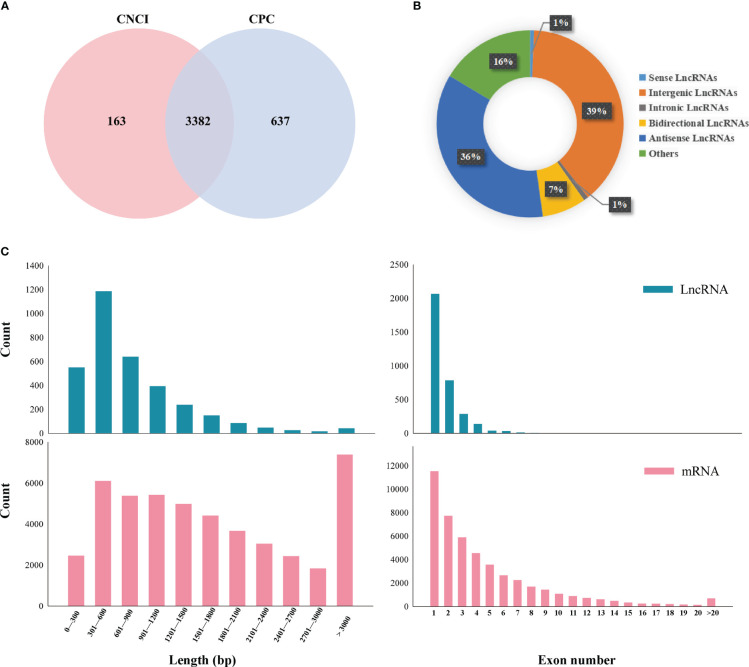
Identification and characterization of lncRNAs with different plumule-vernalization treatments. **(A)** CNCI and CPC software performed Venn map analysis on the identified lncRNAs. **(B)** Proportions of different kinds of lncRNAs. **(C)** Comparison of exon numbers and transcript lengths between lncRNAs and mRNAs.

CNCI and CPC programs were used to evaluate the coding potential of transcripts, and 3382 lncRNAs were identified in the sequenced samples (**Figure 2A**; **Supplementary Table 6**). The lncRNAs were distributed on 10 chromosomes in Chinese cabbage genome the maximum number of lncRNAs was distributed on chromosome A09, and the minimum number of lncRNAs was distributed on chromosome A10 (**Supplementary Figure 2D**). The identified lncRNAs were divided into intergenic lncRNAs, intronic lncRNAs, bidirectional lncRNAs, antisense lncRNAs, and other lncRNAs (uncertain type). The most abundant type was intergenic lncRNAs (39%), followed by antisense lncRNAs (36%), and the least abundant type was internal lncRNAs (1%) (**Figure 2B**; **Supplementary Table 7**). Comparing the expression levels of lncRNAs and mRNAs according to the FPKM values at different treatment points, the expression of lncRNAs was lower than that of mRNA, regardless of whether Chinese cabbage experienced plumule-vernalization (**Supplementary Figures 2A–C**). The abundance of lncRNAs was much smaller than that of mRNAs. To further study the characteristics of the identified lncRNAs, their lengths and exon numbers were analyzed by comparing them with mRNAs. lncRNAs longer than 1200 nt accounted for 18%, while 59% of the mRNAs were shorter than 1000 nt. In addition, there were no lncRNAs with more than 10 exons, but 10% of mRNAs had more than 10 exons (**Figure 2C**).

### LncRNAs and mRNAs in Chinese cabbage were selectively expressed at five stages during plumule-vernalization

**Figure 3:**
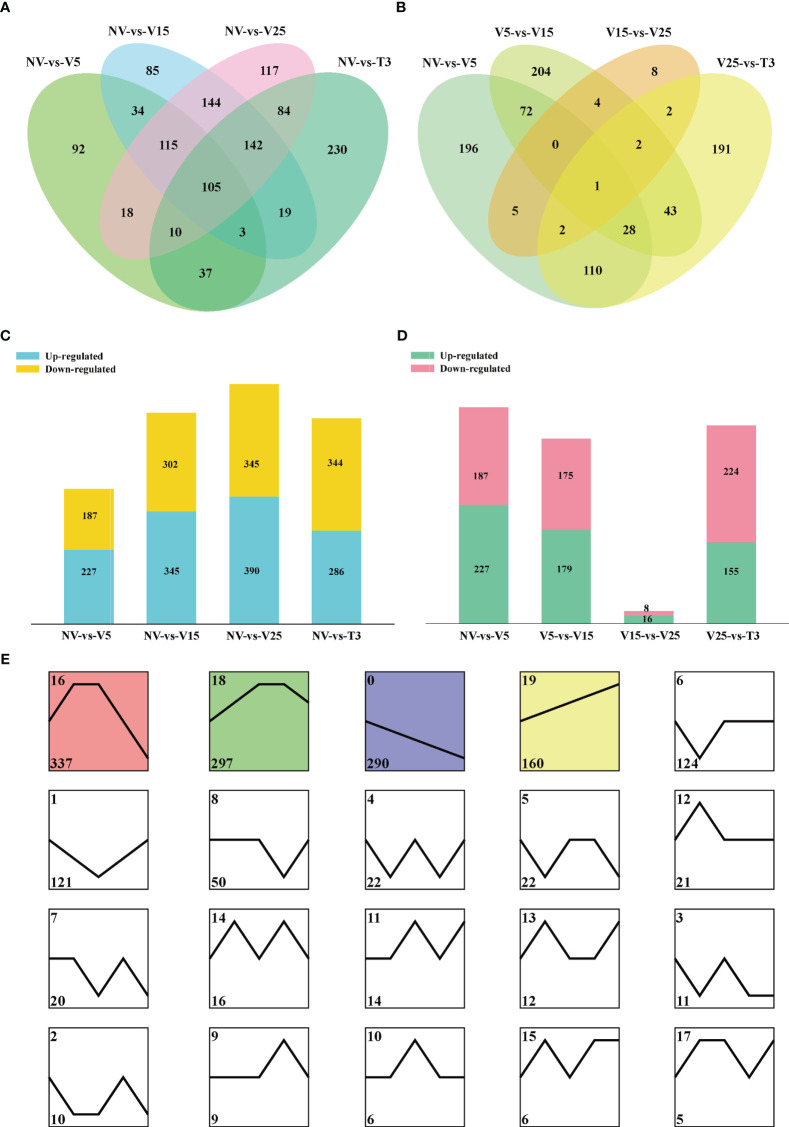
Differentially expressed (DE) lncRNA characteristics in different comparison groups. **(A, C)** The number of DE lncRNAs and their Venn diagrams between NV-vs-V5/V15/V25/T3. **(B, D)** The number of DE lncRNAs and their Venn diagrams between NV-vs-V5, V5-vs-V15, V15-vs-V25, and V25-vs-T3. **(E)** The expression patterns of DE lncRNAs were identified by STEM clustering. The square represents an expression mode, the black lines represent the model expression mode, the number in the upper left corner represents the cluster ID, and the number in the lower left corner represents the number of lncRNAs enriched in the cluster.

DE lncRNAs were determined by comparing vernalized samples to NV samples and two samples from adjacent periods (**Figures 3A, B**). There were 105 common DE lncRNAs in all vernalization samples compared with NV samples (**Figure 3A**). There was only one common DE lncRNA in adjacent samples, *MSTRG*.*25380*.*3* (**Figure 3B**), and the sequencing results showed that there was no gene targeted to it (**Supplementary Table 7**). All treatment points showed similar numbers of up- and downregulated lncRNAs (**Figures 3C, D**). Interestingly, in the V15-vs-V25 samples, 16 DE lncRNAs were upregulated, and 8 DE lncRNAs were downregulated (**Figure 3D**), which was consistent with the SAM phenotypes and flowering days of these two periods. To some extent, V15 had the most dominant vernalization effect. V25-vs-T3 had 155 upregulated lncRNAs and 224 downregulated lncRNAs (**Figure 3D**), indicating that the expression of some genes recovered after the environment was recovered at an appropriate temperature.

Cluster analysis of DE lncRNAs was performed using the STEM program. The 1553 DE lncRNAs were classified into 20 distinct clusters, each representing a group of genes with the same expression pattern (**Figure 3E**). Among them, four clusters were significantly enriched (P < 0.05) with different expression trends according to the background color. Based on the KEGG pathways and GO analysis, target genes of clusters 0, 16, 18, and 19 were annotated (**Supplementary Figure 3**; **Supplementary Table 8**). Cluster 0 enriched genes were continuously downregulated during vernalization, and clusters 16 and 18 were first upregulated and then downregulated. The following categories were enriched in clusters 0, 16, and 18: ‘flavonoid biosynthesis,’ ‘phenylpropanoid biosynthesis,’ ‘starch and sucrose metabolism,’ ‘carotenoid biosynthesis,’ and ‘biosynthesis of secondary metabolites’ (**Supplementary Figures 3A–C**). Vernalization affected the expression of genes in these pathways, which may be involved in vernalization effects. The genes of cluster 19 were continuously upregulated during vernalization and enriched to pathways such as ‘glycine, serine, and threonine metabolism,’ ‘glyoxylate and dicarboxylate metabolism,’ and ‘circadian rhythm-plant’ and GO terms related to the biosynthesis of brassinosteroids (**Supplementary Figure 3D**). Vernalization was also a low-temperature effect, and the upregulation of the expression of these pathway genes, as well as brassinosteroid synthesis and metabolism genes, may enable resistance to the low-temperature effect.

### Interaction network construction of anti-, cis-, and trans-regulated protein-coding genes of DE lncRNAs

**Figure 4:**
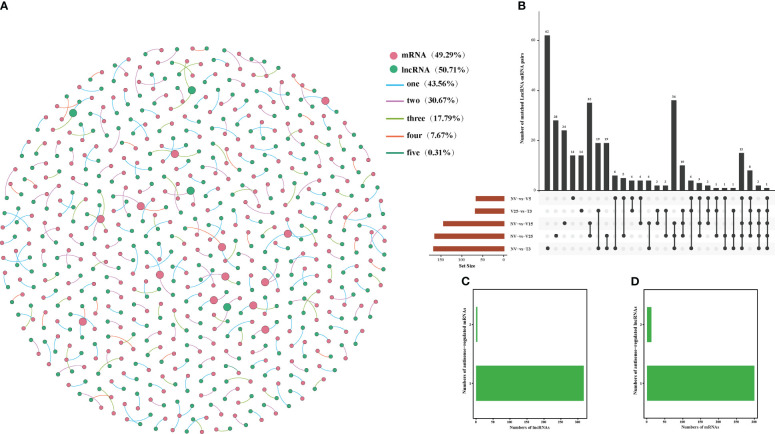
Interactions between DE lncRNAs and anti-regulated DE mRNAs during plumule-vernalization in Chinese cabbage. **(A)** The DE lncRNA–mRNA interaction network of five comparisons (NV-vs-V5, NV-vs-V15, NV-vs-V25, NV-vs-T3, and V5-vs-T3). The nodes represent the mRNAs (red) and lncRNAs (green). The size of the nodes is positively related to the number of mRNAs (lncRNAs) associated with lncRNAs (mRNAs). The color of the line indicates that a relationship exists in several comparison groups (1/2/3/4/5). **(B)** Venn diagram showing the number of matched lncRNA–mRNA pairs in different comparisons. **(C)** The number of DE mRNAs regulated by DE lncRNAs. **(D)** The number of DE lncRNAs that have potential anti-regulatory effects on DE mRNAs.

Correlation analysis between lncRNAs and mRNAs could be conducted in three ways: base complementary pairing between lncRNAs and mRNAs, the regulation of the transcription of protein-coding genes adjacent to lncRNAs, or correlation analysis between lncRNAs and their co-expressed protein-coding genes. LncRNAs regulate the expression of reverse, proximal, and distal protein-coding genes through anti-, cis-, and trans-regulated modes of action (Guil and Esteller, 2012; Fatica and Bozzoni, 2014; Zhao et al., 2018). In our study, we constructed a co-expression analysis using Gephi software according to the expression level of DE lncRNA and DE mRNAs in NV vs V5, NV vs V15, NV vs V25, NV vs T3, and V25 vs T3 and predicted anti-, cis-, and trans-regulated genes. A total of 322 predicted DE lncRNAs matched 313 DE mRNAs with anti-acting effects (**Figure 4**; **Supplementary Table 9**). Among these, 142 anti-regulated matches (43.56%) were exclusively expressed in one comparison group. Only one anti-regulatory match (*MSTRG*.*27145*.*1* match *BraA09g005340*.*3C*) was shared in all comparisons (**Figures 4A, B**; **Supplementary Table 9**-1). NV-vs-V25 had the most matched pairs, and NV-vs-T3 had the least matched pairs, which showed that the longer the vernalization time, the more differential the matched pairs, and some matched pairs recovered after the recovery temperature. Four lncRNAs were co-expressed with two mRNAs, and the remaining lncRNAs had one anti-regulated target (**Figure 4C**; **Supplementary Table 9**-2). Only 4% of the mRNAs corresponded to two lncRNAs, and the remaining mRNAs may have been regulated by one lncRNA (**Figure 4D**; **Supplementary Table 9**-3).

**Figure 5:**
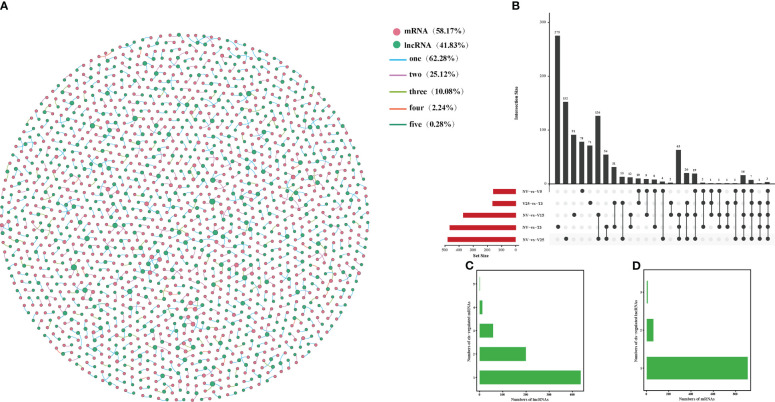
Interactions between DE lncRNAs and cis-regulated DE mRNAs during plumule-vernalization in Chinese cabbage. **(A)** The DE lncRNA–mRNA interaction network of five comparisons (NV-vs-V5, NV-vs-V15, NV-vs-V25, NV-vs-T3, and V5-vs-T3). The nodes represent the mRNAs (red) and lncRNAs (green). The size of the node is positively related to the number of mRNAs (lncRNAs) associated with lncRNAs (mRNAs). The color of the line indicates that a relationship exists in several comparison groups (1/2/3/4/5). **(B)** Venn diagram showing the number of matched lncRNA–mRNA pairs in different comparisons. **(C)** The number of DE mRNAs regulated by DE lncRNAs. **(D)** The number of DE lncRNAs that have potential cis-regulatory effects on DE mRNAs.

The lncRNAs located within 10 kb upstream or downstream of a gene may intersect with cis-acting mRNA, thus participating in the process of transcriptional regulation. It was predicted that 709 DE lncRNAs matched to 986 DE mRNA, having a potential cis-acting effect, while only 3 matching pairs (0.28%) were shared in all comparisons (**Figure 5**; **Supplementary Table 11**). Unlike anti-regulated matches, NV-vs-V15 had the most cis-regulated matching pairs, which were the same as anti-regulated matches, and NV-vs-T3 had the least-matched pairs (**Figure 5B**; **Supplementary Table 11**-**1**). More than 10% of the lncRNAs in all matches had cis-regulated mRNAs, and the remaining lncRNAs targeted one or two mRNAs (**Figure 5C**; **Supplementary Table 11**-2). There were 11 mRNAs corresponding to three lncRNAs, and the highest was 94% of mRNAs corresponding to one lncRNA (**Figure 5D**; **Supplementary Table 11**-**3**).

**Figure 6:**
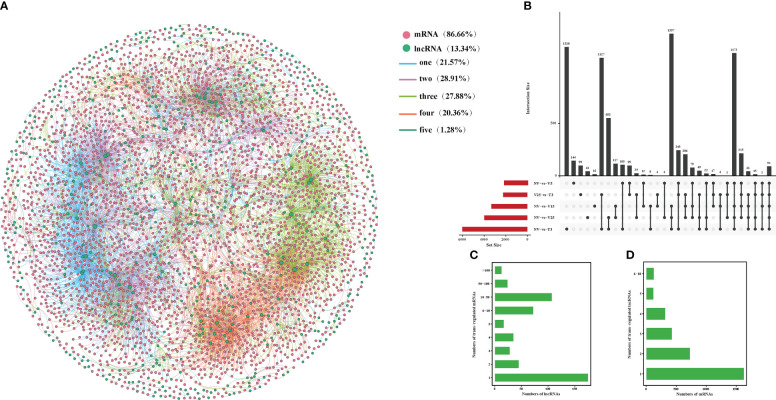
Interactions between DE lncRNAs and trans-regulated DE mRNAs during plumule-vernalization in Chinese cabbage. **(A)** The DE lncRNA–mRNA interaction network of five comparisons (NV-vs-V5, NV-vs-V15, NV-vs-V25, NV-vs-T3, and V5-vs-T3). The nodes represent the mRNAs (red) and lncRNAs (green). The size of the node is positively related to the number of mRNAs (lncRNAs) associated with lncRNAs (mRNAs). The color of the line indicates that a relationship exists in several comparison groups (1/2/3/4/5). **(B)** Venn diagram showing the number of matched lncRNA–mRNA pairs in different comparisons. **(C)** The number of DE mRNAs regulated by DE lncRNAs. **(D)** The number of DE lncRNAs that have potential trans-regulatory effects on DE mRNAs.

The basic principle of trans-target gene prediction was that the function of lncRNAs was not related to the location of coding genes but to the protein-coding genes they co-expressed. Of 7102 pairs, 516 were predicted to have potential trans-acting effects on 3353 DE mRNAs (**Figure 6**; **Supplementary Table 13**). Among the three regulation modes, the maximum shared among all comparisons was the trans-regulatory match, with 91 pairs (**Figure 6B**; **Supplementary Table 13**-1). The number of mRNAs in lncRNA trans-regulation ranged from 1 to 198. More than 66% of the lncRNAs were co-expressed with only one mRNA, and 13 lncRNAs had more than 100 trans-regulated targets. There were 1 to 198 mRNA subjected to trans-regulation of lncRNA. More than 66% of the lncRNAs were co-expressed with only one mRNA, and 13 lncRNAs had more than 100 trans-regulated targets (**Figure 6C**; **Supplementary Table 13**-2). In addition, 1–10 mRNAs corresponded to lncRNAs, of which *BraA05g035260*.*3C* corresponded to 10 lncRNAs, and more than 48% of mRNAs corresponded to only one lncRNA (**Figure 6D**; **Supplementary Table 13**-3).

### Functional enrichment analysis of genes regulated by DE lncRNAs *via* anti-, cis-, and trans-regulatory activity

**Figure 7:**
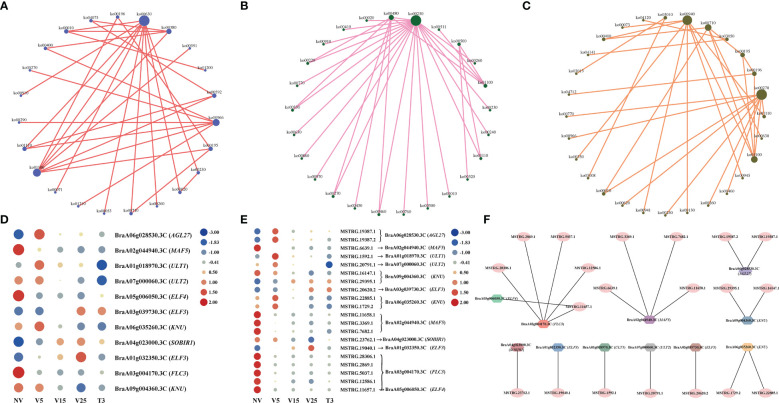
Analysis of lncRNAs and their target genes involved in ‘flowering.’ **(A)** KEGG enrichment analysis of DE lncRNA–anti-regulated genes. **(B)** KEGG enrichment analysis of DE lncRNA–cis-regulated genes. **(C)** KEGG enrichment analysis of DE lncRNA–trans-regulated genes. **(D)** Heat map showing the expression pattern of ‘flowering’-related genes. **(E)** Heat maps showing the expression pattern of lncRNA regulating the ‘flowering’ genes. **(F)** Interaction network between lncRNAs and ‘flowering’ genes.

We conducted KEGG analysis on predicted anti-, cis-, and trans-regulated genes, and drew network diagrams based on the pathway information of gene enrichment and the interaction between different pathways (**Figures 7A–C**). In the KEGG analysis of predicted anti-regulated genes, ko00630 (glycoxylate and dicarboxylate metabolism), ko01100 (metabolic pathways), and ko00966 (glucosinolate biosynthesis) were the three pathways that interacted most with the other pathways (**Figure 7A**; **Supplementary Table 10**). Ko00250 (alanine, aspartate, and glutamate metabolism), ko00480 (glutathione metabolism), and ko01100 (metabolic pathways) were the most associated pathways in the KEGG enriched set of cis-regulated genes (**Figure 7B**, **Supplementary Table 12**). The KEGG enriched set of trans-regulated genes had the most associations with other pathways: ko00270 (cysteine and methionine metabolism), ko00940 (phenylpropanoid biosynthesis), and ko01100 (metabolic pathways) (**Figure 7C**, **Supplementary Table 14**). This indicates that plumule-vernalization causes a large number of metabolic and biosynthetic pathways and that these enriched genes match DE lncRNAs, specifically participating in the regulation of plumule-vernalization.

Through functional annotation and GO analysis of anti-, cis-, and trans-regulated genes, we found 11 genes related to ‘flowering,’ namely, three MADS protein genes (*BraA06g028530*.*3C*, *AGL27*; *BraA02g044940*.*3C*, *MAF5*; *BraA03g004170*.*3C*, *FLC3*), three early flowering protein genes (*BraA05g006050*.*3C*, *ELF4*; *BraA03g039730*.*3C*, *ELF3*; *BraA01g032350*.*3C*, *ELF3*), two late flowering protein genes (*BraA09g004360*.*3C*, *KNU*; *BraA06g035260*.*3C*, *KNU*), and three GO annotation regulation of flower development genes (*BraA01g018970*.*3C*, *ULT1*; *BraA07g000060*.*3C*, *ULT2*; *BraA04g023000*.*3C*, *SOBIR1*) (**Figure 7D**), and drew the expression heat map in the process of plumule-vernalization. The expression of these ‘flowering’ genes had obvious differential changes in the plumule-vernalization process. It was speculated that these flowering genes were affected by vernalization to regulate flowering. The expression amount of lncRNAs matched with these ‘flowering’ genes in the plumule-vernalization process and was made into a heat map (**Figure 7E**). *FLC3* (*BraA03g004170*.*3C*) matched five lncRNAs, and the expression level gradually decreased with vernalization, with significant differences. Interestingly, *ELF4* (*BraA05g006050*.*3C*) and *FLC3* (*BraA03g004170*.*3C*) shared the same lncRNA (*MSTRG*.*11657*.*1*). *AGL27* expression was anti-regulated by two lncRNAs, in which *MSTRG*.*19387*.*1* also trans-regulated *AGL27* (**Figure 7F**). Four lncRNAs were trans-regulated *MAF5*, among which *MSTRG*.*6639*.*1* also anti-regulated *MAF5* (**Figure 7F**). The same lncRNA may regulate different mRNAs, and the same lncRNA can also have different regulatory functions on the same mRNA.

### ceRNA network analysis revealed the miRNA–lncRNA–mRNA network

**Figure 8:**
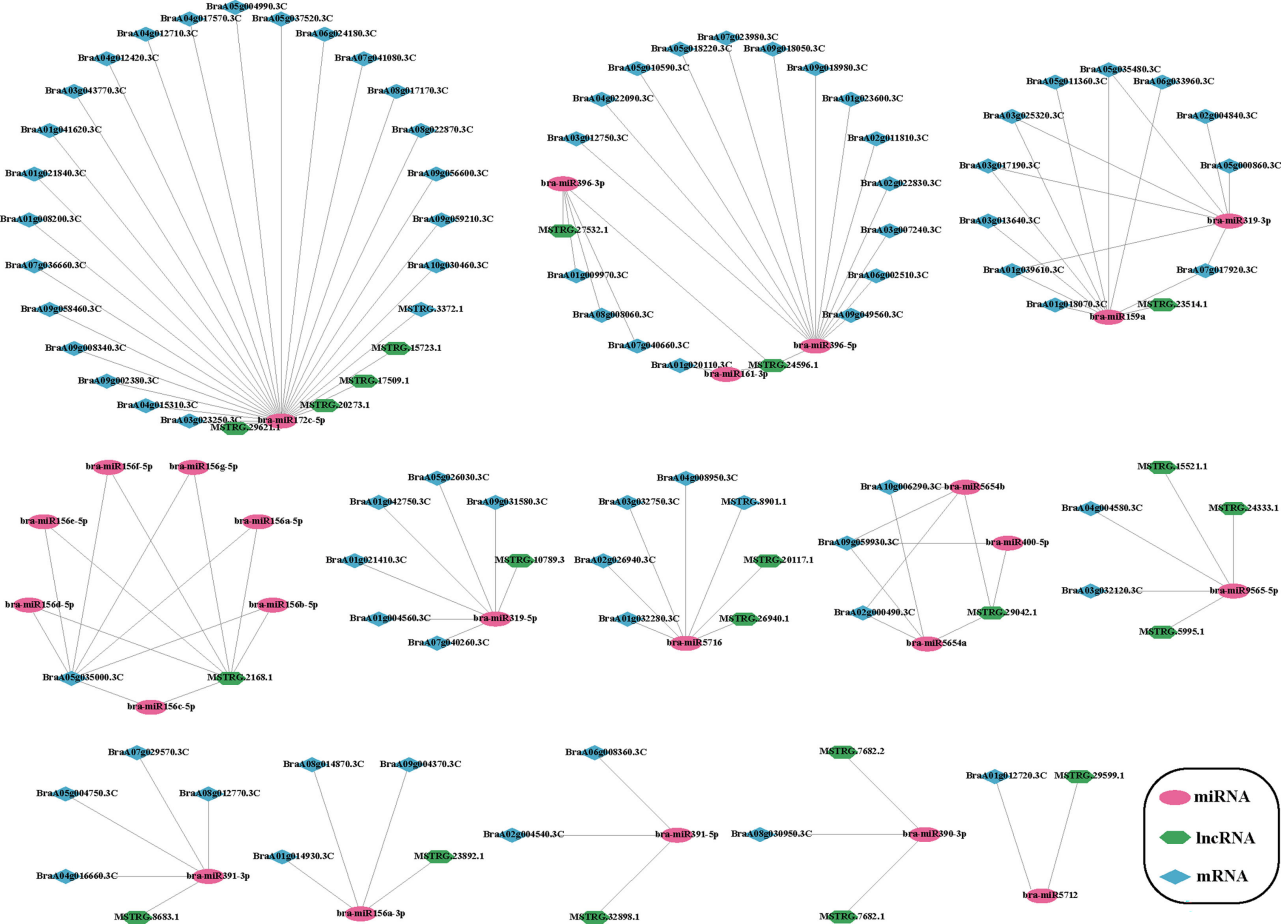
Plumule-vernalization response ceRNA network of Chinese cabbage. The red oval, purple hexagon, and blue diamond nodes represent miRNAs, DE lncRNAs, and DE mRNAs, respectively.

LncRNAs can interact with miRNAs as competitive endogenous RNA and participate in the expression and regulation of target genes. LncRNAs, such as ceRNA and miRNA, can regulate each other. Therefore, we constructed a ceRNA network of Chinese cabbage to predict the interaction among lncRNAs, miRNAs, and mRNAs during plumule-vernalization. Here, we obtained miRNAs from the miRbase database and used them as bait to predict ceRNAs and target mRNAs. Finally, 280 ceRNA pairs were predicted (**Supplementary Table 15**). An interaction network diagram of DE lncRNA and DE mRNA in the predicted ceRNAs was constructed (**Figure 8**). Interestingly, bra-miR156c-5p, bra-miR156d-5p, bra-miR156e-5p, bra-miR156f-5p, bra-miR156g-5p, bra-miR156a-5p, and bra-miR156b-5p were bra-miR156 miRNA types, together with *MSTRG*.*2168*.*1* lncRNA, in coordination with 20 mRNAs (**Supplementary Table 15**). Among them, bra-miR172c-5p formed 80 ceRNA network lines (**Supplementary Table 15**), and 32 showed differential expression. In addition, a few DE mRNAs and DE lncRNAs corresponded to multiple miRNAs, such as *BraA05g035000*.*3C* and *MSTRG*.*2168*.*1* (**Figure 8**). The lncRNAs interacting with these miRNAs might also play an important role in regulating the vernalization-induced flowering of Chinese cabbage.

### LncRNA-*COOLAIR* cooperates with the flowering gene *FLC* to regulate Chinese cabbage’s response to plumule-vernalization

**Figure 9:**
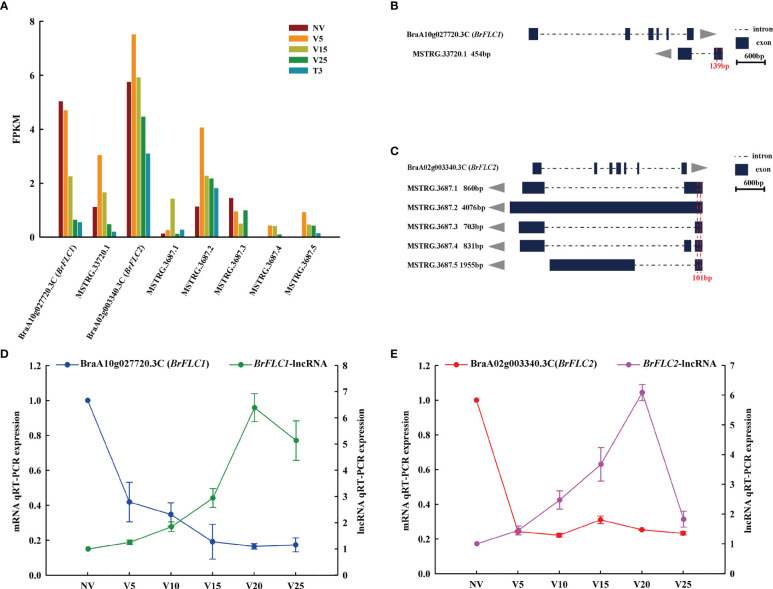
Analysis of lncRNAs and their target *FLC* genes. **(A)** FPKM values of lncRNAs and their target *FLC* genes in the sequenced samples. **(B)** Schematic diagram of the genomic location and transcription direction for lncRNAs and their target *FLC1* gene. Specific primers (red dotted line overlapping regions) were designed for qRT-PCR. **(C)** Schematic diagram of the genomic location and transcription direction for lncRNAs and their target *FLC2* gene. Specific primers (red dotted line overlapping regions) were designed for qRT-PCR. **(D)** Relative expression levels of anti-function lncRNAs and their target *FLC1* gene in all plumule-vernalization samples. Data are presented as the mean ± standard error (SE). **(E)** Relative expression levels of anti-function lncRNAs and their target *FLC2* gene in all plumule-vernalization samples. Data are presented as the mean ± standard error (SE).

The important developmental transformation of plants is from vegetative growth to reproductive growth and gradually from bolting to flowering. Several ways to control flowering would jointly regulate the expression of floral reporter FLOWERING LOCUS C (*FLC*), and vernalization would accelerate the reduction of *FLC* expression, thus promoting plant bolting and flowering (Michaels and Amasino, 1999; Sheldon et al., 1999; Sheldon et al., 2000). *COOLAIR* is a group of antisense lncRNAs expressed from the *FLC* locus that mediates *FLC* expression in plants (Liu et al., 2010; Marquardt et al., 2014; Wang et al., 2014). From our data, we found six antisense lncRNAs targeting *BraA10g027720*.*3C* (*BrFLC1*) and *BraA02g003340*.*3C* (*BrFLC2*). *MSTRG*.*33720*.*1* targeted *BrFLC1* (**Figures 9A, B**), and *MSTRG*.*3687*.*1*, *MSTRG*.*3687*.*2*, *MSTRG*.*3687*.*3*, *MSTRG*.*3687*.*4*, and *MSTRG*.*3687*.*5* targeted *BrFLC2* (**Figures 9A, B**). According to the RNA-seq data, *BrFLC1* expression gradually decreased with vernalization, and the expression of its matching lncRNA (*MSTRG*.*33720*.*1*) was highest at V5 and then decreased. *BrFLC2* first rose and then declined during vernalization, and its five matching lncRNAs, *MSTRG*.*3687*.*1* and *MSTRG*.*3687*.*2*, also rose first and then declined. The expression of *MSTRG*.*3687*.*3* in vernalization was lower than that in non-vernalization. *MSTRG*.*3687*.*4* and *MSTRG*.*3687*.*5* had increased expression levels due to vernalization, and NV was not expressed (**Figure 9A**). We performed qRT-PCR verification on *BrFLC* and its matching lncRNAs. Because the structures of the five lncRNAs matching *BrFLC2* were very similar, we selected the common region for qRT-PCR primer design, as shown in **Figures 9B, C** (red label, 139 bp and 101 bp). The expression of *BrFLC1* and *BrFLC2* and their matching lncRNAs showed the opposite pattern (**Figures 9D, E**), and the expression of *BrFLC* decreased with vernalization, and the expression of *BrFLC1* lncRNA increased up to V20 and decreased to V25. The expression patterns of *BrFLC2* and *BrFLC2*-lncRNA with vernalization were the same as those of *BrFLC1* and *BrFLC1*-lncRNA.

### Expression verification of lncRNAs and their potential target genes

**Figure 10:**
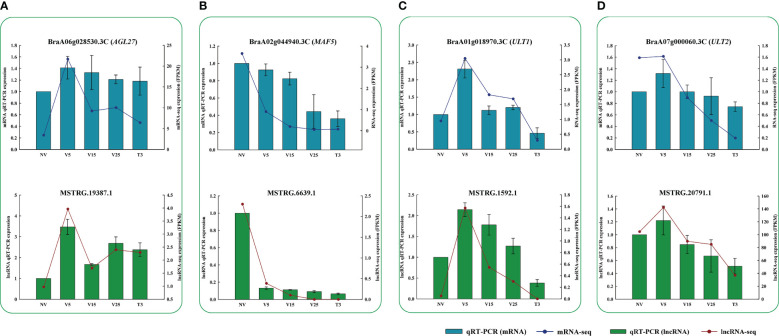
Verification of RNA-seq results *via* qRT-PCR. Fragment per kilobase of transcript per million mapped reads (FPKM) and relative expression levels of lncRNAs and their regulated mRNAs. **(A)** lncRNA and the *AGL27* gene. **(B)** lncRNA and the *MAF5* gene. **(C)** lncRNA and the *ULT1* gene. **(D)** lncRNA and the *ULT2* gene. Data are presented as the mean ± standard error (SE).

According to our analysis, we performed qRT-PCR verification on some lncRNAs related to flowering-targeted genes, as shown in **Figure 10**. The results of qRT-PCR were consistent with those obtained from RNA-seq, indicating that these identified lncRNAs might regulate a series of genes with different functions through co-expression to respond to plumule-vernalization in Chinese cabbage.

## Discussion

Although great progress has been made in vernalization genes and noncoding RNAs in *Arabidopsis* (Swiezewski et al., 2009; Helliwell et al., 2011; Heo and Sung, 2011; Sun et al., 2013b; Kim et al., 2017b; Zhao et al., 2018), research on noncoding RNA in the vernalization of Chinese cabbage has also made progress (Li et al., 2016; Zhang et al., 2022). However, the mechanism of vernalization affecting noncoding RNA and promoting the flowering of Chinese cabbage is still poorly understood. Whole-genome gene expression profiles may enable insight into the plumule-vernalization of Chinese cabbage. To better understand the response to plumule-vernalization at the transcription level, we evaluated the transcriptional landscape of a Chinese cabbage variety (JHX) that needs vernalization for bolting and flowering using high-throughput RNA-seq. Chinese cabbage is a crop with plumule-vernalization and seedling-vernalization (Yu et al., 2016). Plumule-vernalization means that germinated seeds can sense low temperatures, and after plumule-vernalization, through artificial treatment or natural low temperatures, plumules can enter bolting, flowering, and fruiting without the heading stage. Moreover, Chinese cabbage ‘JHX’ is a DH line, and selecting pure line material can ensure smooth sequencing and analysis.

The SAM status of many plants and various characteristics have been observed by slices to further determine the growth and development period of plants and the function of genes (Takagi and Ueguchi, 2012; Cheng et al., 2018; Nie et al., 2021; Olas et al., 2021). We used toluidine blue to dye the SAM of Chinese cabbage under different plumule-vernalization treatments combined with different flowering times after plumule-vernalization and selected representative periods NV, V5, V15, V25, and T3 for RNA-seq (**Figure 1**). The general characteristics of lncRNAs in Chinese cabbage during plumule-vernalization treatment were described in detail in this study. CNCI and CPC programs identified 3382 lncRNAs in the sequencing samples, and most of the lncRNAs were shorter than 1000 nt in length and contained far fewer exons than mRNA (**Figure 2**), which was consistent with the previous results of Chinese cabbage lncRNA response to heat stress (Song et al., 2021).

Interestingly, in the DE lncRNA analysis, V15 vs V25 only upregulated 16 lncRNAs and downregulated 8 lncRNAs, which was the control group with the least DE lncRNAs (**Figure 3D**). We also found that the flowering days at this stage were very similar after V15 to V25 treatment (**Figure 1C**). The vernalization effect should have a critical value, and after reaching the critical value, the effect of vernalization promoting rapid bolting and flowering can be maintained.

We constructed a co-expression network of predicted anti-, cis-, and trans-regulated genes and their matching DE lncRNAs (**Figures 4**–**6**). We found 11 genes related to ‘flowering’ through functional annotation and GO analysis of anti-, cis-, and trans-regulated genes and 20 matching lncRNAs (**Figures 7D–F**). Phylogenetic analysis places *FLC* in a subfamily with two other MADS-box genes, *AGL27* and *AGL31* (Alvarez-Buylla et al., 2000). *AGL27* is a regulator of flowering time and a repressor of the transition from vegetative development to reproductive development, and *AGL27* has also been named *FLOWERING LOCUS M* (*FLM*) (Scortecci et al., 2001). In this study, the expression of *AGL27* first increased and then decreased rapidly during plumule-vernalization, and the two matching lncRNAs (*MSTRG*.*19387*.*1* and *MSTRG*.*19387*.*2*) showed the same expression change. Moreover, *AGL27* expression was anti-regulated by two lncRNAs, in which *MSTRG*.*19387*.*1* also trans-regulated *AGL27*. *MAF5* is normally repressed, and its ectopic overexpression under non-inductive short days causes late flowering (Kim and Sung, 2010). In our study, *MAF5* was also affected by vernalization. With vernalization, the expression of *MAF5* was significantly reduced. There were three trans-regulated *MAF5* in the four matching lncRNAs, and *MSTRG*.*6639*.*1* not only anti-regulated *MAF5* but also trans-regulated *MAF5*. Some studies have shown that when *BrFLC1*, *BrFLC2*, and *BrFLC3* were transformed into *Arabidopsis*, the transformed plants showed delayed flowering time (Kim et al., 2006). Five lncRNAs trans-regulated *FLC3* in our study, and the expression levels decreased significantly with vernalization. EARLY FLOWERING 3 (*ELF3*) and *ELF4* were first identified on genetic screens for photoperiodism mutants and were found to regulate circadian rhythms (Hicks et al., 1996; Hicks et al., 2001; Doyle et al., 2002). Mutants *elf3* and *elf4* promote the early flowering of *Arabidopsis* (Doyle et al., 2002; Yoshida et al., 2009; Natsui et al., 2010). In our study, the expression of two lncRNAs was trans-regulated, and two *ELF3* genes gradually increased with the vernalization process. Compared with previous results (Doyle et al., 2002; Yoshida et al., 2009; Natsui et al., 2010), it is possible that *ELF3* genes are not affected by vernalization and promote the early flowering of Chinese cabbage. However, the expression of *MSTRG*.*11657*.*1* and its trans-regulated *ELF4* gradually decreased during vernalization, and *MSTRG*.*11657*.*1* was also an lncRNA of trans-regulated *FLC3*. It was thus clear that *ELF4* (*BraA05g006*,*050*.*3C*) may be affected during vernalization as *FLC3* because of the reduced expression during vernalization, which promotes flowering. *KNUCKLES* (*KNU*) mediates the repression of *WUSCHEL* (*WUS*) in floral meristem determinacy control and terminates the floral meristem (Asbe et al., 2015; Nemhauser and Torii, 2016). In this study, four lncRNAs trans-regulated two *KNU* genes. The expression of *KNU* (*BraA06g035260*.*3C*) increased first from NV to V5 but decreased rapidly from V5 to T3, and two matching lncRNAs (*MSTRG*.*22885*.*1* and *MSTRG*.*1729*.*2*) had the same expression mode. The expression of *KNU* (*BraA09g004360*.*3C*) decreased due to vernalization, but the expression of its two matching lncRNAs (*MSTRG*.*16147*.*1* and *MSTRG*.*29395*.*1*) was opposite; one was affected by vernalization, and its expression was increased, and the other was decreased. *Arabidopsis* trxG genes *ULTRAPETALA1* (*ULT1*) and *ULT2* together restrict shoot and floral stem cell activity and establish apical–basal gynoecium polarity (Monfared et al., 2013). The two DE lncRNAs, *MSTRG*.*1592*.*1* and *MSTRG*.*20791*.*1*, cis-regulated *ULT1* and *ULT2*, respectively, and their expression levels increased from NV to V5 and decreased from V5 to T3, indicating that the *ULT* genes and their matching lncRNAs may be affected by vernalization to promote the transition of the growth period. The *SUPPRESSOR OF BIR1 1* (*SOBIR1*) in *Arabidopsis* was annotated as a negative regulation of floral organization abscission (Leslie et al., 2010). The expression of the *SOBIR1* gene was gradually upregulated during the vernalization of Chinese cabbage, while the expression of *MSTRG*.*23762*.*1*, an lncRNA of trans-regulated *SOBIR1*, was downregulated with vernalization. Whether the change in *SOBIR1* expression is caused by vernalization remains to be studied. These candidate lncRNAs and their mRNA related to ‘flowering’ were affected by plumule-vernalization. These findings improved our understanding of how vernalization promotes flowering in Chinese cabbage, and these candidate lncRNAs and mRNA could be used in future studies.

*BrFLC1*, *BrFLC2*, and *BrFLC3* are copies that are syntenic to *AtFLC*, which originated from the genome triplication event of Chinese cabbage since it diverged from *Arabidopsis* (Wang et al., 2011). *COOLAIR* is an lncRNA that plays an important role in regulating *FLC* during vernalization in *Arabidopsis* (Hawkes et al., 2016). Three types of antisense intragenic RNA *COOLAIR* in *Arabidopsis* are divided into two categories: proximally polyadenylated class I and distally polyadenylated class II (Swiezewski et al., 2009; Hornyik et al., 2010). Previous studies detected only *COOLAIR*-like transcripts detected from *BrFLC2*, which were involved in the suppression of *BrFLC2* (Li et al., 2016). In our study, six antisense lncRNAs targeted *BrFLC1* (*BraA10g027720*.*3C*) and *BrFLC2* (*BraA02g003340*.*3C*) (**Figure 9A–C**). Interestingly, MSTRG.33720.1 anti-regulated *BrFLC1*, which was not identified in previous studies. At the same time, the lncRNAs of five anti-regulated *BrFLC2* were structurally different from those previously studied (Li et al., 2016). Li et al. grouped them into two classes (class I and class II) according to polyadenylation sites in intron 6 or within the promoter of *BrFLC2* (Li et al., 2016). In this study, four lncRNAs of anti-regulated *BrFLC2* were all class II types (cleared and polyadenylated within the sequence antisense to the *BrFLC2* promoter) (**Figure 9C**). To define the relationship between *BrFLC1*, *BrFLC2*, and their matching lncRNAs, the abundance of related transcripts was analyzed using real-time PCR. The expression of *BrFLC1* decreased with vernalization, but the expression of *BrFLC1*-lncRNA increased gradually and decreased slightly at V25 (**Figure 9D**). *BrFLC2* decreased rapidly at the beginning of vernalization and maintained a low expression pattern. The expression of *BrFLC2*-lncRNA increased gradually and decreased significantly at V25 (**Figure 9E**).

## Conclusions

As a vernalization vegetable crop, a few reports have identified long noncoding RNAs under vernalization in Chinese cabbage. Hence, we identified lncRNAs in the SAM of Chinese cabbage on a large scale and identified lncRNAs under plumule-vernalization treatment. This study expands our knowledge of lncRNAs involved in the plumule-vernalization response by identifying DE lncRNAs and conducting anti-, cis-, and trans-functional analyses in Chinese cabbage. Many genes and lncRNAs that promoted the bolting and flowering of Chinese cabbage, as well as antisense lncRNAs, targeted *BrFLC* differently from previous studies. The critical lncRNAs identified in our study provide valuable information for plumule-vernalization response lncRNAs in Chinese cabbage and provide rich resources for further research on the biological functions of lncRNAs in plants.

## Data availability statement

The original contributions presented in the study are publicly available. These data can be found here: NCBI, PRJNA921831.

## Author contributions

SJZ, HZ, and RS conceived and designed the experiments; YD and XG performed the experiment; YD and GL wrote the manuscript; SW, ZL, and CS analyzed and submitted the data; SFZ, FL, and ZF modified the manuscript. All authors contributed to the article and approved the submitted version.

## Supplementary material

Supplementary Figure 1 Sample correlation heat map and Pearson correlation analysis (PCA) among all 15 libraries. **(A, B)** LncRNA sample correlation and PCA. **(C, D)** mRNA sample correlation and PCA.

Supplementary Figure 2 Comparison of features of lncRNAs and mRNAs during plumule-vernalization in Chinese cabbage. **(A)** Violin plot of log_10_ (FPKM) for mRNA. **(B)** Violin plot of log_10_ (FPKM) for lncRNAs. **(C)** Comparing the cfeatures of lncRNAs and mRNAs by bean plot. **(D)** Percentage of lncRNAs on each chromosome in Chinese cabbage.

Supplementary Figure 3 KEGG and GO analyses of DEGs in significant clusters. **(A)** KEGG and GO analysis of DEGs in cluster 0. **(B)** KEGG and GO analysis of DEGs in cluster 16. **(C)** KEGG and GO analysis of DEGs in cluster 18. **(D)** KEGG and GO analysis of DEGs in cluster 19.
