# Supplementary figures and images for: Identification of long noncoding RNAs involved in plumule-vernalization of Chinese cabbage
Source: Front Plant Sci. 2023 Mar 14;14:1147494. doi: 10.3389/fpls.2023.1147494 (PMC10043383; doi:10.3389/fpls.2023.1147494)

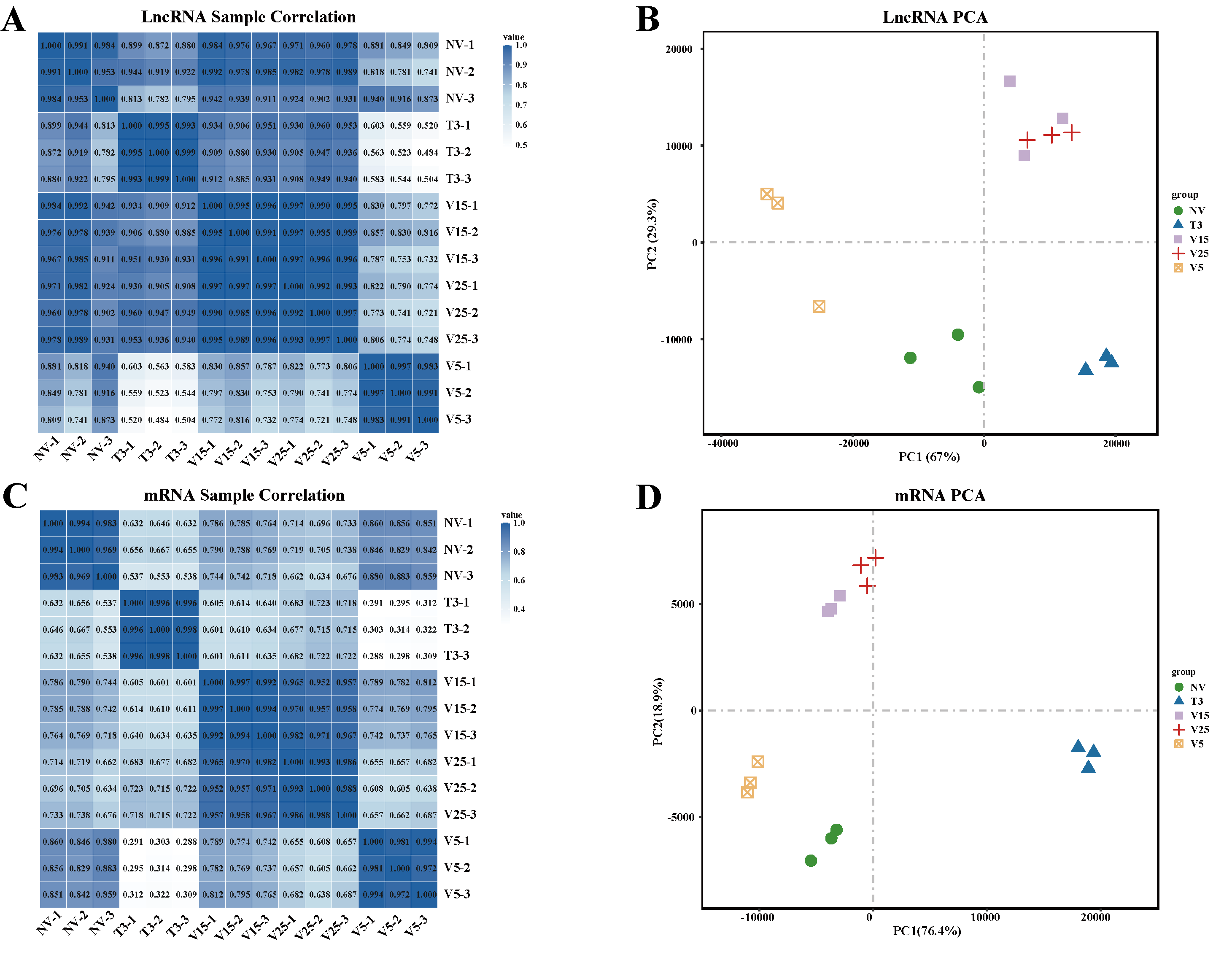

Supplement: Supplementary Figure 1 — Sample correlation heat map and Pearson correlation analysis (PCA) among all 15 libraries. (A, B) LncRNA sample correlation and PCA. (C, D) mRNA sample correlation and PCA. [file Image_1.jpeg]

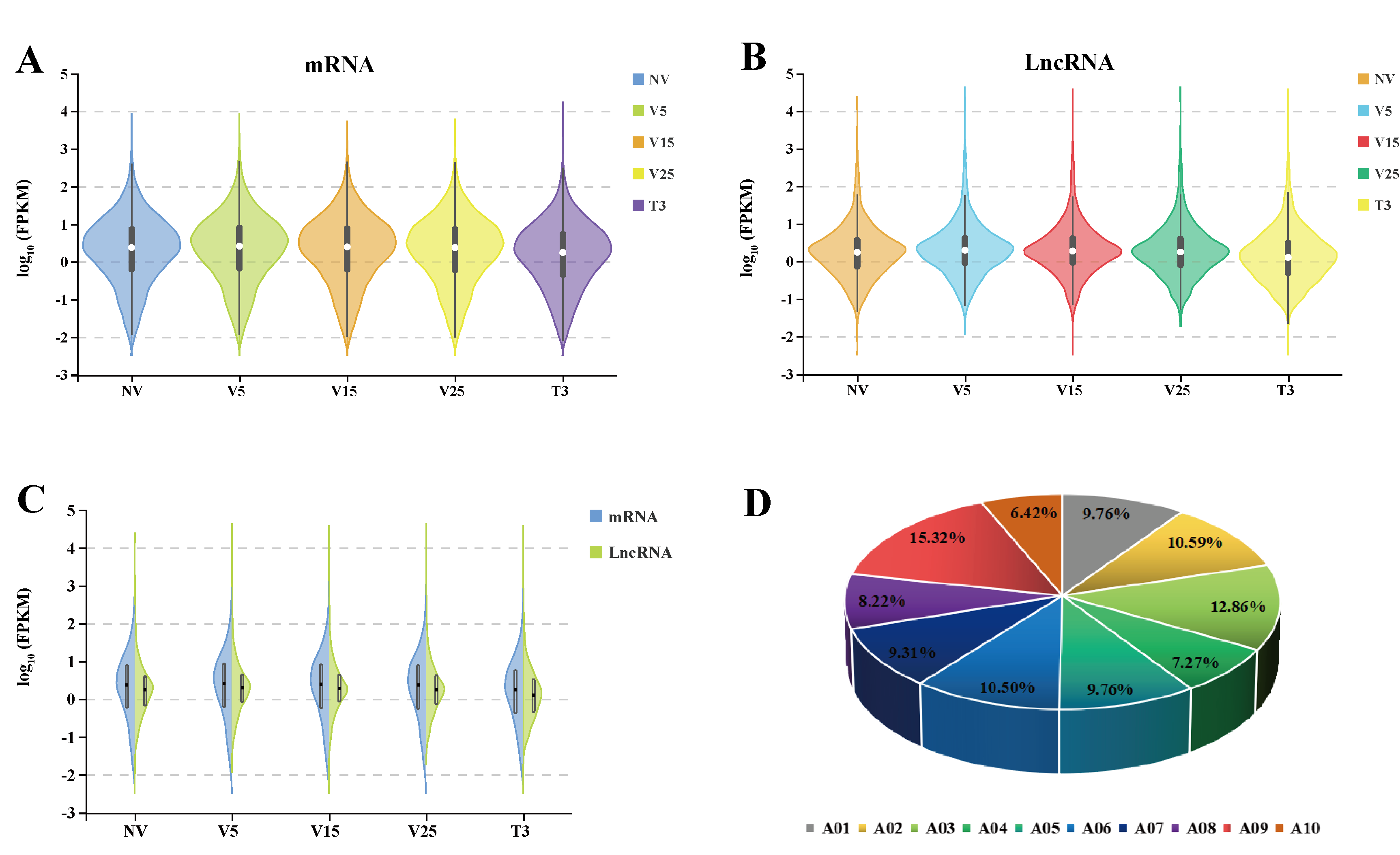

Supplement: Supplementary Figure 2 — Comparison of features of lncRNAs and mRNAs during plumule-vernalization in Chinese cabbage. (A) Violin plot of log10 (FPKM) for mRNA. (B) Violin plot of log10 (FPKM) for lncRNAs. (C) Comparing the cfeatures of lncRNAs and mRNAs by bean plot. (D) Percentage of lncRNAs on each chromosome in Chinese cabbage. [file Image_2.jpeg]

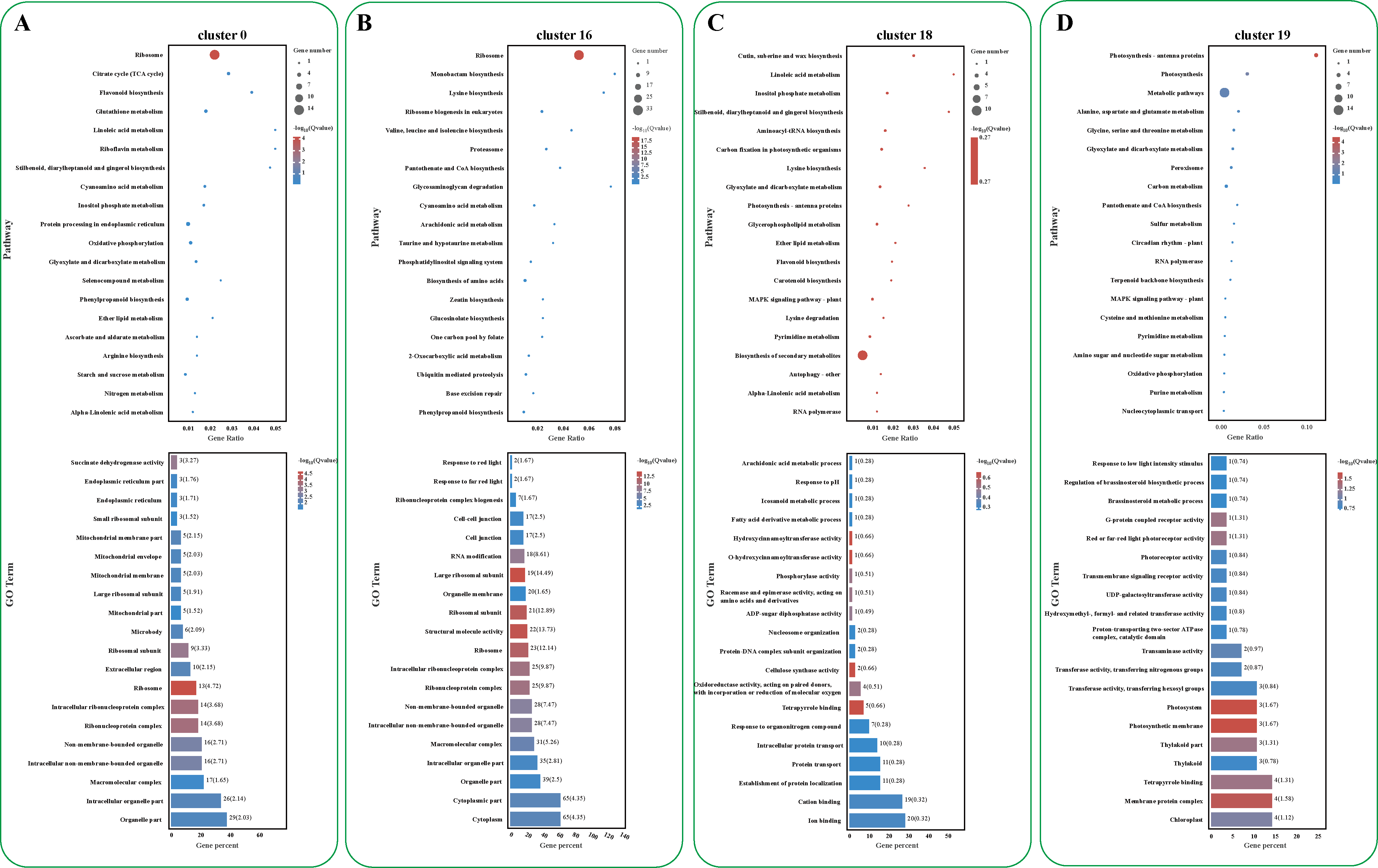

Supplement: Supplementary Figure 3 — KEGG and GO analyses of DEGs in significant clusters. (A) KEGG and GO analysis of DEGs in cluster 0. (B) KEGG and GO analysis of DEGs in cluster 16. (C) KEGG and GO analysis of DEGs in cluster 18. (D) KEGG and GO analysis of DEGs in cluster 19. [file Image_3.jpeg]
